# Campylobacteriosis, Shigellosis and Salmonellosis in Hospitalized Children with Acute Inflammatory Diarrhea in Georgia

**DOI:** 10.3390/pathogens11020232

**Published:** 2022-02-10

**Authors:** Maia Metreveli, Salome Bulia, Iamze Shalamberidze, Liana Tevzadze, Shota Tsanava, Juan Cruz Goenaga, Kerstin Stingl, Paata Imnadze

**Affiliations:** 1 Faculty of Medicine, Ivane Javakhishvili Tbilisi State University, 0179 Tbilisi, Georgia; pimnadze@ncdc.ge; 2 Department of Gastroenteric Infection Diseases, Tbilisi Children Infectious Diseases Clinical Hospital, 0171 Tbilisi, Georgia; salomeabulia@yahoo.com (S.B.); Iashalamberidze@hotmail.com (I.S.); 3 General Bacteriology Laboratory, Richard Lugar Public Health Research Center in Tbilisi, National Center for Disease Control and Public Health, 0198 Tbilisi, Georgia; l_tevzadze@yahoo.com (L.T.); sh.tsanava@ncdc.ge (S.T.); 4 National Reference Laboratory for Campylobacter, Department of Biological Safety, German Federal Institute for Risk Assessment, 12277 Berlin, Germany; juan-cruz.goenaga@bfr.bund.de; 5 National Center for Disease Control and Public Health, 0198 Tbilisi, Georgia

**Keywords:** *Campylobacter*, gastroenteritis, diarrheal illness, enteropathogens, selective media, epidemiological pyramid, children, Georgia

## Abstract

This is the first study on campylobacteriosis carried out in Georgia. It targeted 382 hospitalized children with acute inflammatory diarrhea. The study was conducted between July 2020 to July 2021 based on the main infection clinic of the capital city. Culture-based bacteriological methods were followed by phenotypic and Real-time PCR tests for bacterial confirmation and identification. The data revealed recent epidemiologic prevalences of the three main causative bacteria in the target population. *Shigella sonnei* with 19.1% (95% CI: 15.2–23.4%) was the most frequently detected pathogen followed by *Campylobacter* spp. with 12.3% (95% CI: 9.2–16.0%) and *Salmonella* spp. with 4.9% (95% CI: 3.0–7.6%). However, in 63.6% of the samples, the causative agent remained unknown. Species differentiation of *Campylobacter* spp. revealed 81% *Campylobacter jejuni* and 19% *Campylobacter coli*. An epidemiological pyramid with estimated magnification factors may give more insights into the burden of campylobacteriosis among the studied population, resulting in a putative annual incidence of 6 per 1000 children in Tbilisi. Children with campylobacteriosis were younger (median age 40 months (interquartile range (IQR) 22−95)) than with shigellosis (median age 92 months (interquartile range (IQR) 52−140)). However, no statistically significant difference was found with the age range of patients with campylobacteriosis and salmonellosis as well as with salmonellosis and shigellosis. In conclusion, *Campylobacter* spp. may be suspected to be the second most frequent bacterial causative agent of acute inflammatory diarrhea in hospitalized children and the primary cause in the 0–3 age group in Georgia. In addition, *Campylobacter* CROMagar showed better selectivity in comparison to mCCDA selective agar of stool samples in our study.

## 1. Introduction

*Campylobacter* is the leading food-borne bacterial pathogen, causing gastroenteritis worldwide. Based on the reported number of campylobacteriosis cases, the “true incidence” is estimated to annually affect between 4.4 and 9.3 human beings per 1000 population. However, the number of cases in Low and Middle-income countries (LMIC) is largely unknown [1]. *Campylobacter* is often found in poultry and cattle; other animals, including young dogs and cats, other pets, pigs and birds, may also be reservoirs of *Campylobacter,* capable of infecting humans [2].

Infection with *Campylobacter* cause acute, mostly self-limited gastrointestinal illness characterized by diarrhea, fever and abdominal cramps [3]. In principle, the symptoms of *Campylobacter* infection are mostly indistinguishable from acute gastrointestinal infections caused by *Salmonella, Shigella* and *Yersinia*. Clinical outcomes in most patients are reported as watery and bloody diarrhea and increased bowel movements per day during acute illness [4]. The most important treatment comprises the maintenance of hydration and electrolyte balance. Most patients with *Campylobacter* infection have a self-limited illness for 3 to 7 days [5]. There are, however, severe cases with a need for antibiotic treatment, which include patients with high fevers, bloody stools, prolonged illness, pregnancy and immunocompromised states [6,7,8]. For example, in around 30% of the reported cases in Germany, antibiotic treatment, in particular, fluoroquinolones and macrolides, were used [9]. In addition, *Campylobacter* infections can cause long-term autoimmune sequelae, such as reactive arthritis, irritable bowel syndrome and the Guillain-Barré syndrome. The results indicated that *Campylobacter* may account for up to 41% of all cases of Guillain-Barré syndrome [10].

In high-income countries, monitoring campylobacteriosis cases is most often realized by reporting laboratory-diagnosed infections in a population. It critically depends on the diagnostic methods implemented in the health care system. While laboratory confirmation of enteric infections is routinely performed in industrialized countries, most LMIC lack routine diagnostics. Indeed, in particular, surveillance data on *Campylobacter* are scarce. Preliminary data indicate that, in Asian countries, campylobacteriosis in children is among the five most frequent diarrheal illnesses, even after accounting for some presence of *Campylobacter* in controls [1,11]. A multisite cohort study of enteropathogens in children < 2 years of age conducted at low-resource settings revealed high *Campylobacter* prevalences associated with growth shortfalls. Factors associated with a reduced risk of *Campylobacter* detection included exclusive breastfeeding, drinking water treatment, improved latrines and macrolide antibiotic treatment [12]. Another study showed higher *Campylobacter* abundance in the fecal microbiota of children < 1 year of age in Africa, associated with breastfeeding. Although most of the *Campylobacter* were *C. jejuni* or *C. coli*, a novel *Campylobacter* species was proposed to occur in infants and an association between breastfeeding and *Campylobacter* infection suggested selection for *C. jejuni* and *C. coli* strains unable to metabolize fucose [13].

In LMIC, systematic surveillance of enteric pathogens is hampered by an insufficient number of microbiological laboratories, a potential lack of supplies and equipment and the challenge of coordinating monitoring efforts across sectors. Therefore, the clinical significance of these infections and their prevalence in different populations remains unclear [1]. 

Georgia has a geographically important location within Europe, bridging North and South Caucasus. The country has simplified entry regimes with neighboring countries, some Middle Eastern and Asian countries and also benefits from visa liberalization with Europe and other European Neighbourhood Policy (ENP) countries. In addition, international traveler trips in Georgia have been rapidly increasing in recent years. In 2017, they reached a record number of 7.9 million two times exceeding the local population [14]. The demographics of the country deviate from other European countries, such as France, Italy and Germany, in that the proportion of young people is higher. Likewise, the Georgian population displayed a median age of 37.0 in 2020, and children compose 20% of the population [15], while in the same year the French, Italian and German population, were in median 42.3, 47.3 and 45.7 years old, respectively. 

Data on infectious diseases were obtained from the National Center for Disease Control and Public Health (NCDC) of Georgia www.ncdc.ge (accessed on 24 January 2022) [16]. Up to now, in total, seven single cases of campylobacteriosis were reported between 2012 and 2020 for the age group of children, corroborating the need for improved surveillance of this fastidious bacterium within the Georgian public health system (Table 1). 

One of the reasons might be that microbiological laboratories in hospitals do not routinely analyze *Campylobacter* spp. while performing stool cultures for other enteric pathogens. Therefore, neither incidence nor burden of campylobacteriosis in Georgia is known. Meanwhile, the official statistics of “Gastroenteritis of Presumed Infectious Origin and Foodborne Diseases” in the country remain alarmingly high, particularly in the children population [17]. As shown in Table 1, in more than 92% of the cases, the etiological agent of the disease was not identified and was recorded as “Diarrhea and Gastroenteritis of Presumed Infectious Origin”. *Shigella sonnei* has remained a leading causative bacteria for more than two decades. Meanwhile, data for the remaining bacteria seem to be highly underreported. 

Our study intended to deliver the first systematic prevalence data of campylobacteriosis among hospitalized children with acute inflammatory diarrhea and to reveal the recent epidemiologic trends in comparison to the currently known enteric bacteria *Salmonella* and *Shigella*.

## 2. Results

In total, 382 stool samples were collected between July 2020 and July 2021 from hospitalized children with acute inflammatory diarrhea and gastroenteritis. 

### 2.1. Prevalences of the Three Analysed Diarrhea-Causing Bacteria, Age Distribution of the Patients and Seasonality

**Figure 1:**
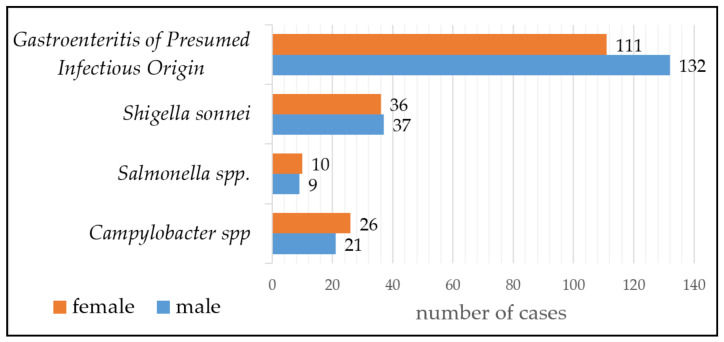
Prevalence of the three main enteropathogenic bacteria in 382 stool samples of children with acute diarrhea and patients’ sex distribution.

From the 382 stool samples, 139 specimens (36.4%) were positive for any of the three tested enteric pathogens (Figure 1). There were no samples with mixed infection. 

The age of the patients varied from 2 months up to 211 months (~17 years). The median age of all patients was 52 months, while 71 months was the median of the subset of patients with the identified pathogen. 

Among enteropathogenic bacteria, *Shigella sonnei* was the most common (19.1% (95% CI: 15.2–23.4%) *n* = 73)*,* followed by thermophilic *Campylobacter* spp. (12.3% (95% CI: 9.2–16.0%) *n* = 47) and *Salmonella* spp. (4.9% (95% CI: 3.0–7.6%) *n* = 19). However, in more than half of the cases (*n* = 243, 63.6%), the etiological agent was not detected (Figure 1). 

Phenotypic tests with the BioMerieux API system and a Real-time PCR (Mayr et al., 2010) confirmed *Campylobacter* spp. from the suspected colonies in 47 cases, from which 81% (*n* = 38) were identified as *C. jejuni* and 19% (*n* = 9) as *C. coli*. BioMerieux E20 tests system confirmed 19 positive samples for *Salmonella* spp. and 73 for *Shigella* spp. from the suspected colonies grown on selective Hektoen and XLD plates. Furthermore, polyvalent antisera tests identified all *Salmonella* spp. as C-group strains and *Shigella* spp. as *Shigella sonnei*.

The patients were hospitalized either with an acute hemocolitis with bloody diarrhea and high fever for three days or with prolonged diarrhea containing water, mucus and blood. The average hospitalization time was 5 days. Almost all patients had the following symptoms: hemocolitis, fever, abdominal pain, tenesmus and rarely vomiting. 55% of the patients were girls *n* = 26, 45% *n* = 21 boys. 

**Figure 2:**
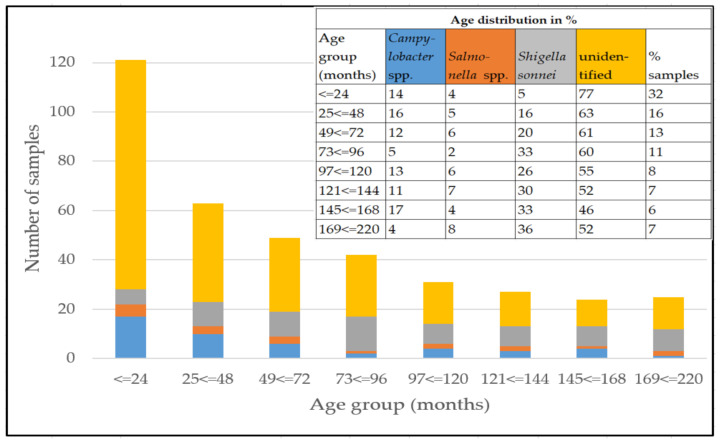
Age distribution of the patients and identified pathogens in stool samples. The graph shows absolute numbers, while a fraction of pathogen detection in distinct age groups is depicted in the inserted table (in %). Blue, *Campylobacter* spp.; orange, *Salmonella* spp.; grey, *Shigella sonnei*; yellow, unidentified pathogen.

The patients age group distribution of the sample set categorized by pathogen detection is illustrated in Figure 2. Thirty-two percent of the patients were up to 2 years old, and 61% were preschoolers.

**Figure 3:**
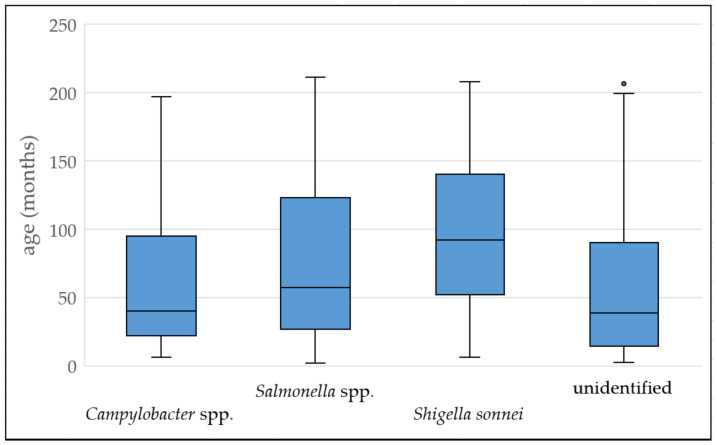
Boxplots of age distribution (in months) of patients with campylobacteriosis, salmonellosis, shigellosis and unidentified pathogen; horizontal line, median age; IQR were calculated with the inclusion of the median.

The distribution of the patients’ ages with *Campylobacter* spp. infection (median 40 months (interquartile range (IQR) 22−95)) was significantly different compared to patients infected with *Shigella sonnei* (median 92 months (interquartile range (IQR) 52−140)) using the Mann–Whitney test with U = 994 and *p* < 0.001 (Figure 3). However, the difference of the patient age with campylobacteriosis was not significantly important from that with salmonellosis (median age 57 months (interquartile range (IQR) 27−123)), U = 374, *p* = 0.3), and of patients with salmonellosis compared to shigellosis (U = 534, *p* = 0.124).

We further analyzed whether any seasonality was observed for enteric pathogen infection in children in Tbilisi. For this purpose, the prevalence was categorized by month of sampling.

**Figure 4:**
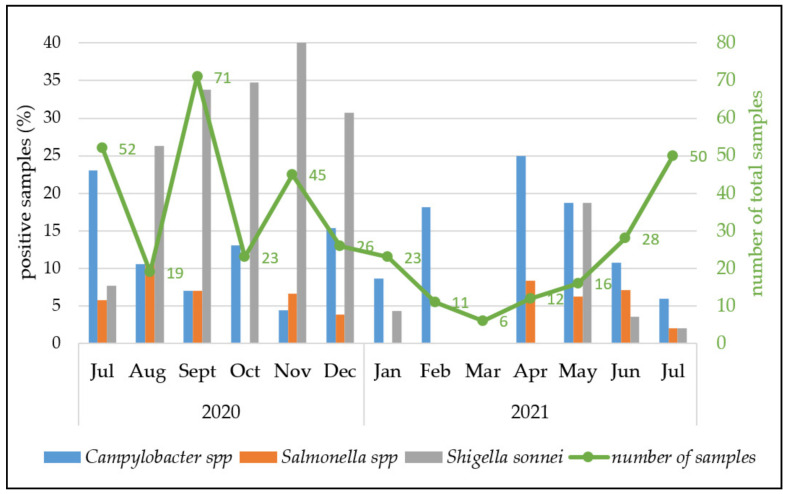
Number of samples tested (right *y*-axis, green) and fraction of positive samples per identified enteropathogens by month (left *y*-axis, black).

As depicted in Figure 4, there was no obvious seasonal difference in the number of detected campylobacteriosis and salmonellosis cases, although the overall number of samples was low. In contrast, shigellosis appeared to be predominant during autumn until the beginning of winter (Figure 4). It is noted that almost all patients were citizens of Tbilisi, where the seasonal temperature is significantly different. 

**Figure 5:**
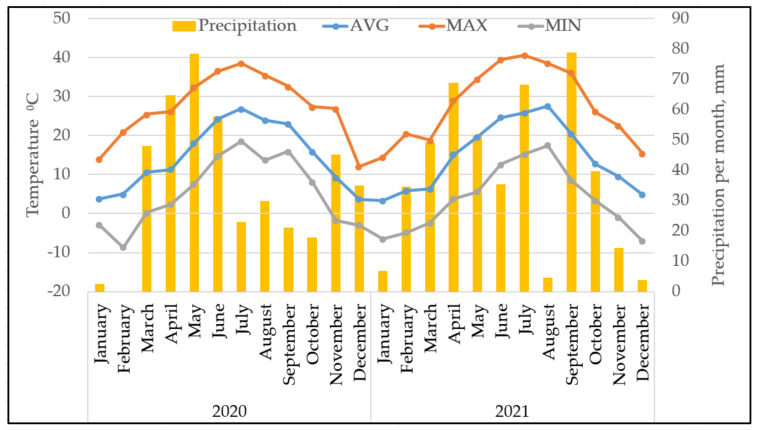
Air temperature and monthly precipitation in Tbilisi in 2020 and 2021 [18]; AVG, average; MAX, maximal; MIN, minimal temperature.

Tbilisi has a moderately warm climate, transitioning from steppe to humid subtropics. It is characterized by mild winters and summers. In 2020 and 2021, the average annual temperature was 14.7 °C (Figure 5). The absolute minimum temperature was −8.7 °C in February 2020. The annual precipitation was 424 mm and 456 mm in 2020 and 2021, respectively.

### 2.2. Estimated Burden of Campylobacteriosis 

**Figure 6:**
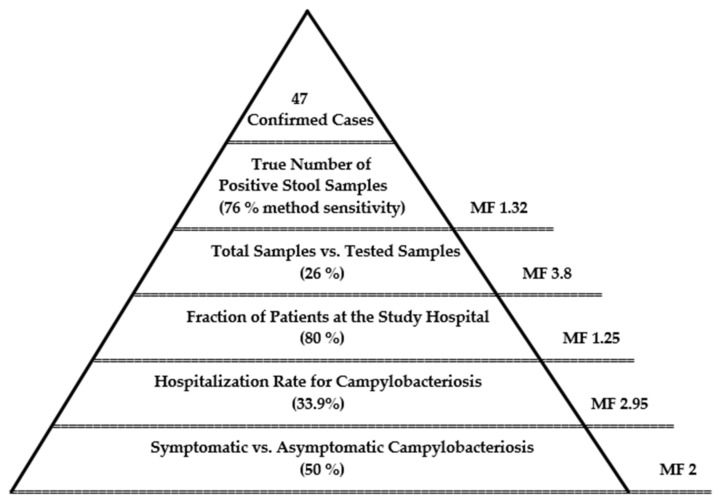
Epidemiological pyramid of campylobacteriosis for the estimation of the true number of campylobacteriosis cases among children in Tbilisi.

An epidemiological pyramid was set in a way to extrapolate the results of the study to the Tbilisi children’s population in order to estimate the “true prevalence” of campylobacteriosis (Figure 6). Multiplication factors (MFs), a measure of the magnitude of underestimation, were taken directly from the literature or derived from statistical information. The pyramid shows estimated incidence data at five levels: 

1. Symptomatic campylobacteriosis: Although *Campylobacter* is rarely identified in the stools of healthy persons, depending on the population studied, as many as 50% of persons who are infected during outbreaks were asymptomatic [19]. Taking this number into account, the calculated MF is 2.

2. Hospitalization rate of campylobacteriosis: In European countries, the proportion of hospitalized patients due to campylobacteriosis is around 33.9% [20], which derives a MF of 2.95.

3. Fraction of patients at the study hospital: The Tbilisi Children Clinical Hospital of Infectious Diseases is the main hospital for gastroenteric illnesses in the city; therefore, around 80% of children patients were hospitalized at this location, corresponding to an MF of 1.25. 

4. Fraction of tested samples for campylobacteriosis: The annual number of patients at the Tbilisi Children Clinical Hospital of Infectious Diseases with acute bacterial inflammatory diarrhea was 1450, from which 26% (*n* = 382) of the samples were tested for *Campylobacter* spp. in this study (MF 3.8). 

5. Sensitivity of the laboratory method: For the identification of *Campylobacter* from stool samples, we applied the standard culture-based laboratory method, estimated to have a median sensitivity of 76% [21], leading to an MF of 1.32.

6. Laboratory confirmed cases were delivered by the presented study. They constitute the tip of the pyramid. 

To estimate the “true number” of the infection, the number of confirmed cases delivered in the study is multiplied by the MFs indicated above (MFs = 1.32 × 3.8 × 1.25 × 2.95 × 2 = 37). Hence, the “true incidence” of campylobacteriosis among the Tbilisi children’s population might be around 1800 cases annually. Given a total number of 283.400 children in Tbilisi in 2020 (GeoStat 2020, 0–19 Age Group), this refers to 1:1500 cases annually or an incidence of around 6 per 1000. 

### 2.3. mCCDA vs. CHROMagar

ISO 10272-1: 2017 does not demand the use of two selective media. However, dependent on the background microbiota and on the state of the fastidious *Campylobacter* spp., it is well-known that the choice of selective media might influence the effectiveness of detection [22,23]. Thus, we performed a dual selection of *Campylobacter* spp. on either mCCDA or CHROMagar *Campylobacter* in order to increase detection sensitivity, and we identified *Campylobacter* spp. in 47 samples with either mCCDA (*n* = 28) and/or CHROMagar (*n* = 39). Hence, out of 47 positive samples, mCCDA showed false negative results or was overgrown by the background microbiota in 30% (*n* = 12) of the samples; in 7 cases, the medium was not applied. While for *Campylobacter* CHROMagar, false negative results were obtained in 17% (*n* = 8). 

**Figure 7:**
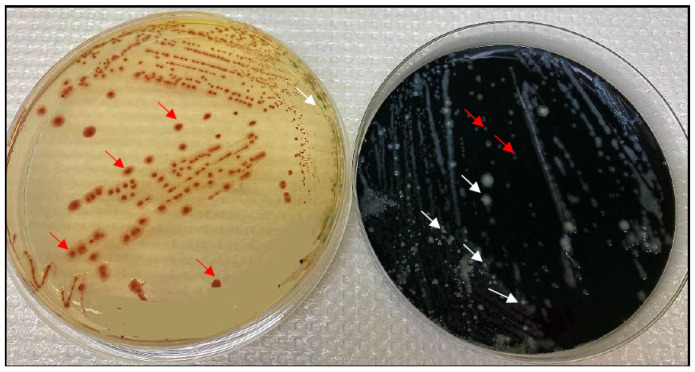
Comparison of *Campylobacter* detection on mCCDA (**right**) and CHROMagar (**left**). The red arrows indicate examples of thermophilic *Campylobacter spp*. colonies, while the white arrows point to background flora colonies. The sample No. on CHROMagar (**left**) was masked by a same-colored region.

The comparison of the performance of both selective media delivered an odds ratio (OR) of 1.4, with a 95% confidence interval (CI) of 1.1 to 1.7, *p* = 0.038, showing a better performance of CHROMagar *Campylobacter* versus mCCDA (Figure 7). The typical greyish metallic colonies of thermophilic *Campylobacter* on mCCDA might be overseen due to dominant background microbiota so that CHROMagar *Campylobacter* appeared to be more selective and easier to handle for the routine laboratory, not necessarily focused only on *Campylobacter* spp. detection. 

## 3. Discussion

Our results showed that campylobacteriosis might be the second leading bacterial cause of acute inflammatory diarrhea in hospitalized children in the country. An interesting yet unexplained finding was that children with *Campylobacter* spp. were significantly younger than children with shigellosis, while the age distribution of children infected with *Salmonella* compared to *Campylobacter* did not differ significantly. Meanwhile, in school-aged children and adolescents, *Shigella sonnei* was dominant. This finding becomes more significant as age differences were previously observed in a recent study from Israel, with more younger patients with campylobacteriosis and salmonellosis than with shigellosis [24]. It may be helpful for quick decision making regarding initial empirical antimicrobial agents, if necessary [25].

Putting our study results with the existing national statistical data in line, we could suppose that campylobacteriosis is the second most frequent causative agent of bacterial gastroenteric illnesses on a country level, and its burden on public health might be largely underestimated. We tried to estimate the burden of the infection by applying the epidemiological pyramid method and adjusting data in order to provide more realistic estimates of *Campylobacter* incidence. This method is widely applied for priority setting for infectious disease in order to account for uncertainties not captured by the surveillance system. 

Due to the scarcity of information for public health systems at low- and middle-income countries such as Georgia, MFs to correct underestimation, taken from other countries, might increase uncertainties. However, for decision-making processes, this approach is crucial, in particular for those countries. In our study, it was estimated that the “true incidence” of campylobacteriosis among Tbilisi children’s population might be over 1800 cases annually. In this study, we focused on *Campylobacter* spp., but the incidence of *Salmonella* and *Shigella* might be underestimated, however, probably to a less extent. Still, in more than half of the samples, the pathogen was unidentified. We suppose that, in the future, enteropathogenic *Escherichia coli* (EPEC) and other bacterial pathogens should also be analyzed as well.

*Campylobacter* was identified among five pathogens considered to contribute to the substantial burden of disease spreading through animal feces in domestic environments in LMICs [26]. Despite the fact that all participants of our study were urban residents, contacts with animal feces, e.g., from domestic pets and street dogs at kids’ playgrounds, could also have contributed to the infection. Thus, there is an urgent need for further epidemiologic studies on food consumption patterns of Georgian children, including breastfed infants, with a focus on the development of campylobacteriosis, which might help in source attribution of this important pathogen. Moreover, large-scale studies are needed to confirm the differences in these clinical findings, considering the relatively low significance noted in a modest number of children. Higher positive rates for bacteria may be related to the use of multiplex PCR tests for detecting bacterial enteropathogens, particularly *Campylobacter* spp., as they are more sensitive than culture methods [27,28]. 

## 4. Materials and Methods

This study was approved by the Institutional Ethics Committee of the Tbilisi Children Infectious Diseases Clinical Hospital in 2020. It was performed at the Tbilisi Children Infectious Diseases Clinical Hospital, which is specialized in infection illnesses. Children with acute diarrhea and gastroenteritis of presumed infectious origin are predominantly directed to this site (80–85% of the cases in the city). For the identification and confirmation of *Campylobacter* species, laboratory capacity was enhanced at the Richard Lugar Public Health Research Center in Tbilisi. In addition, the stool samples were tested at the clinic laboratory for the detection of *Salmonella* and *Shigella*. Additional metadata on patients were collected at the hospital for further epidemiological analysis.

### 4.1. Sample Collection and Transport

In order to guarantee high sensitivity, in particular, for *Campylobacter* spp. detection, samples were transported on Cary-Blair medium (Biolife Italiana S.r.l., Milan, Italy) at cooling temperatures and analyzed within 24 h after sampling. The samples were tested for the three main diarrhea-causing bacterial pathogens, *Salmonella*, *Shigella* and *Campylobacter.*

In total, 382 stool samples were collected from July 2020 to July 2021 from hospitalized children suspected of having acute inflammatory diarrhea and gastroenteritis. The study was designed to include all participants, fulfilling the criterion of acute bacterial diarrhea. However, the sample number was limited because of the availability of participation agreement, a sufficient number of stool samples and working hours of the physicians involved in the sampling, guaranteeing that the samples could be analyzed within 24 h after sampling. Acute inflammatory diarrhea was defined as acute diarrhea accompanied by at least one of the following symptoms and/or laboratory findings: fever with abdominal cramping, nighttime diarrhea, leukocyte-positive stool, acute bloody diarrhea or hemoglobin-positive stool. Clinical information was collected, including hospitalization date, age, sex, disease manifestation and symptoms duration. 

### 4.2. Detection and Phenotypic Identification of Pathogen Species

For *Campylobacter*, the detection was performed according to ISO 10272-1:2017 part C, using *Campylobacter* CROMagar as a second selective media. Less than 20% of the samples were also enriched with Preston broth (Biolife Italiana S.r.l., Milan, Italy) [29], but the results showed no enhanced detection. Incubation was performed at 42 °C in a microaerobic gas mixture consisting of 85% nitrogen, 10% carbon dioxide and 5% oxygen [30]. Modified charcoal, cefoperazone, deoxycholate agar (mCCDA) (Thermo Fisher Specialty Diagnostics Ltd., Hampshire, UK) [31] and Chromogenic agar *Campylobacter* (CHROMagar, France) [32] served as two selective media for the isolation of *Campylobacter* species. Confirmation of suspected colonies grown on the media was performed applying the Biomerieux system ApiCampy (Biomerieux Inc, Marcy-l’Etoile, Lyon, France). 

Hektoen Enteric Agar (Biolife Italiana S.r.l., Milan, Italy) and Xylose Lysine Deoxycholate (XLD) agar (Oxoid, UK) were applied for the selection of *Salmonella* spp. and *Shigella* spp. from the stool samples. The plates were incubated aerobically at 37 °C for 24 h. Growth of the targeted bacteria was detected by evaluating their characteristic appearance on Hektoen agar (small bluish-green colonies and black-centered colonies) and XLD agar (small red colonies and black-centered colonies). Confirmatory identification was conducted through the biochemical reaction patterns using a standard bacterial identification system (API 20E, BioMerieux, Marcy-l’Etoile, Lyon, France) and polyvalent antisera (Biolife Italiana S.r.l., Milan, Italy) [33]. 

### 4.3. Confirmation of Campylobacter Species Differentiation by Real-Time PCR Analysis

Cell material of isolates was resuspended in 5% Chelex 100 resin (Bio-Rad Laboratories GmbH, Feldkirchen, Germany) and heated for 15 min at 95 °C for thermal lysis. Cell debris was centrifuged for 5 min at 14,000× *g*, and the supernatant containing bacterial DNA was used for PCR analysis at a volume of 2.5 µL. For species identification, the real-time PCR method, according to Mayr et al., 2010, was used [34] with the fluorophore combination FAM, JOE, TAMRA and Cy5. Oligos in HPCL-grade at final concentrations of 300 nM (Sigma Aldrich, Steinheim, Germany), 100 nM dark-quenched probes (TIB MOLBIOL, Berlin, Germany) and 1U of Platinum Taq DNA polymerase (Thermo Fisher Scientific Inc., Waltham, Massachusetts, USA) were used. In short, specific fragments of the *mapA*, the *ceuE* and the *gyrA* gene, specific for *C. jejuni*, *C. coli* or *C. lari*, respectively, were targeted. As amplification control, 25 copies of the IPC-ntb2 was applied [35]. 

### 4.4. Data Analysis

Statistical analyses were performed applying SPSS version 15.0 (International Business Machines Corporation (IBM), New York, NY, USA). The Mann–Whitney test was used for comparing the age distribution of the patients infected with either bacterial pathogen. The Odds Ratio was calculated to find the statistical evidence of the efficiency of selective media.

### 4.5. Morbidity Surveillance Pyramid

The morbidity surveillance pyramid is a useful tool to illustrate the availability of morbidity data at each surveillance level. With each ascending level (from the community to healthcare institutions (GPs, hospital, laboratory), to regional and national public health agencies), data availability shrinks and only a fraction of cases from the level below is captured [36].

## 5. Conclusions

In conclusion, there was a high incidence of intestinal campylobacteriosis in hospitalized children in Tbilisi. The present study revealed that *Campylobacter* infection is endemic in the country, and microbiological laboratories should actively pursue the isolation or detection of *Campylobacter* spp. in cases of diarrhea in routine stool cultures. The data are also crucial for epidemiological investigations of the cases with respect to source attribution and infection prevention. We hope that the information will push policy makers to support building clinical laboratories’ capacity for the identification of relevant pathogens, in particular *Campylobacter*, and enhance the national epidemiological surveillance system in the country.

## Figures and Tables

**Table 1:** Numbers of Gastroenteric Infection Diseases in <15 years Age Group in Georgia. Table is Modified, Data from the annual reports of NCDC Georgia, 2021, www.ncdc.ge (accessed on 24 January 2022) [16].

	2012	2013	2014	2015	2016	2017	2018	2019	2020
NT * salmonellosis	76	42	51	24	43	60	102	99	100
Shigellosis	431	107	493	906	391	435	517	267	488
Enterohaemorrhagic Escherichiosis	4	3	2	0	1	2	19	13	26
Campylobacteriosis	3	2	0	-	-	-	-	-	2
Diarrhea and Gastroenteritis of Presumed Infectious Origin	19,305	18,344	18,081	19,841	17,596	10,337	11,963	10,019	7423

* NT, non-typhoidal.

## Data Availability

The data that support the findings of this study are available on request from the corresponding authors.
